# Intussusception in a Routine Colonoscopy

**DOI:** 10.14309/crj.0000000000000422

**Published:** 2020-07-10

**Authors:** Abdelwahab Ahmed, Jinyu Zhang, Kutait Anas

**Affiliations:** 1 Wayne State Medical School, Detroit, MI; 2 Division of Gastroenterology and Hepatology, Henry Ford Hospital, Detroit, MI

## Abstract

We present a 42-year-old woman who developed colo-colonic intussusception of the transverse colon near the hepatic flexure within a few hours after a routine colonoscopy. After conservative management with pain medication and hydration, her symptoms completely resolved within 24 hours. Colonic intussusception after a colonoscopy is rare, and the present case describes the most conservative approach leading to a complete resolution of symptoms.

## INTRODUCTION

An intussusception during or after colonoscopy is an extremely rare occurrence. Only 12 cases could be identified through a thorough PubMed search—2 of which are pediatric cases. Most cases involved a visit to the emergency department with peritoneal signs (i.e., diffuse abdominal pain, vomiting, or nausea), and all but 2 cases (including the present case) required surgical intervention. In every case, the diagnosis was confirmed with a computed tomography scan before an approach was chosen. Of the 2 cases not requiring surgical management, one required endoscopic intervention via colonoscopy, whereas the other had symptom resolution with conservative management only. To our knowledge, this is the only recorded case of colo-colonic intussusception after colonoscopy treated successfully with only pain management and observation. We suggest that conservative management be carefully considered in the setting of similar cases.

## CASE REPORT

A 42-year-old woman with no surgical history and no significant medical history other than diarrhea for 2 months presented to our office with a 3-month history of abdominal pain and diarrhea. The pain was not associated with nausea, vomiting, dysphagia, or blood in the stool. Colonoscopy was performed electively to investigate the cause of her chronic diarrhea.

During the colonoscopy, the quality of the preparation was adequate, and the patient's tolerance of the procedure was appropriate. The terminal ileum was intubated and seemed normal. Four cold forceps biopsies were taken from the ileum. A single sessile polyp, measuring 8-mm in size, was found and removed by cold snare polypectomy from the ascending colon. A single diminutive sessile polyp was removed in the same fashion in the cecum. Random colon biopsies were obtained using biopsy forceps. The pathology report came back negative for inflammatory bowel disease and microscopic colitis. Total cecal intubation time was 4 minutes, and the withdrawal time was 20 minutes. The patient received Meperidine 100 mg intravenously and Midazolam 4 mg intravenously. There was no repositioning or abdominal pressure needed during the procedure.

**Figure 1.**
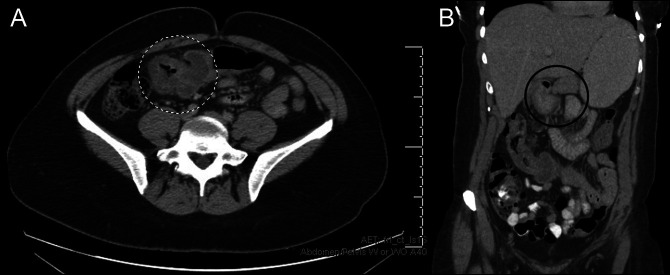
(A and B) Initial abdominal and pelvic computed tomography with contrast showed signs of intussusception of the transverse colon near the hepatic flexure.

**Figure 2.**
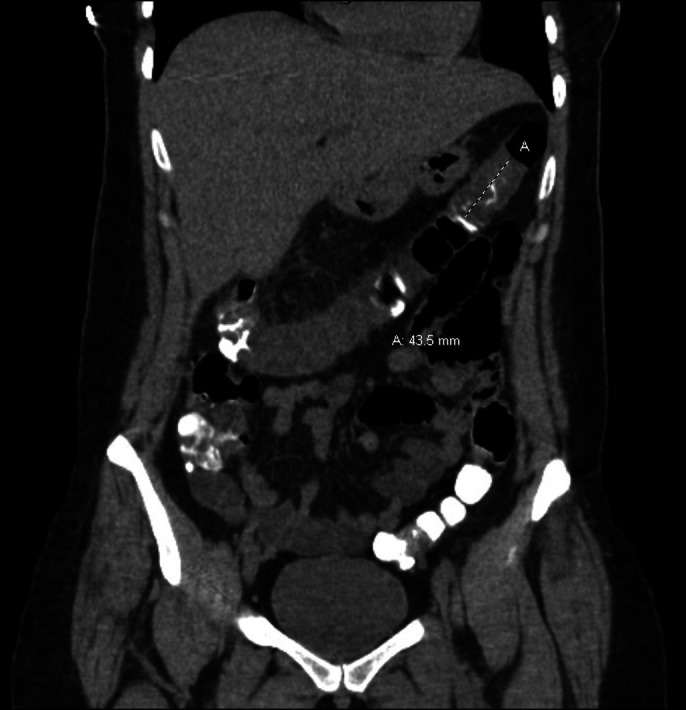
A day after treatment, computed tomography showing resolution of the intussusception.

Later in the day, the patient presented to the emergency department complaining of diffuse epigastric abdominal pain and inability to pass flatus since the colonoscopy. Abdominal and pelvic computed tomography (CT) with contrast showed signs of intussusception of the transverse colon near the hepatic flexure (Figure 1). The patient was admitted for an acute care surgery consultation and was initially managed conservatively with pain medication. The next day, the CT scan showed resolution of the intussusception, and the patient was able to pass stool (Figure 2). The patient was subsequently discharged home with no evidence of recurrence on chart review.

## DISCUSSION

Intussusception is among the rarest causes of bowel obstruction in adults and is much more prevalent in childhood.^1^ In adults, approximately 70%–90% are associated with an identifiable lesion serving as a lead point.^2^ In most cases, this lesion is reflective of an underlying malignancy, which is managed by resection of the intussuscepted bowel. However, colonoscopy-induced intussusception may not need to be treated, similarly, because there is a possibility of spontaneous resolution with conservative management. Other causes of adult intussusception include benign tumors, celiac disease, inflammatory bowel disease, appendicitis, pancreatitis, and foreign bodies, although these account for a much smaller proportion of cases; these cases are managed by surgical resection of the intussuscepted bowel.^3^ Currently, there are a few hypotheses to explain the etiology of colonoscopy-induced intussusception.

A colonoscopy may cause a state of hyperperistalsis in which the colon attempts to vent the insufflated air, leaving it in a state more susceptible to intussusception.^1^ Although it has been well described that carbon dioxide insufflation during colonoscopy is better absorbed by the gastrointestinal mucosa than room air, it has yet to be determined whether the aforementioned venting occurs with both methods, or even, to the same degree. In our case, the colonoscopy was performed with room air. It has also been postulated that during a colonoscopy, the formation of complex loops of bowel may occur and result in a transient intussusception.^2^ One possible mechanism is a “vacuum effect” that may be induced during the concomitant aspiration of gas and retrieval of the colonoscope, which creates the possibility for a segment of proximal bowel to invaginate on a segment of distal bowel.^4,5^

Although the insufflation of the colon during colonoscopies and the methods of scope retrieval may increase the likelihood of intussusception, it has also been hypothesized that the sites of biopsies may serve as lead points for intussusception, as described in 4 cases.^6–9^ However, in the present case, biopsies were taken from the ascending colon and the cecum, whereas the intussusception that followed was present in the transverse colon near the hepatic flexure. Although this would not exclude the aforementioned hypothesis as an explanation for this case, we believe that it would be less likely.

Of the 12 cases reported in the literature, in all but 2 cases, colonoscopy-induced intussusception was managed by bowel resection because this is the standard of care for symptomatic adult intussusception.^10–12^ Given the prevalence of malignant causes for adult intussusception, it is understandable why surgery was so often sought during the cases of colonoscopy-induced intussusception. However, it is crucial to recognize that colonoscopy-induced intussusception occurs because of a different mechanism. In our case, the patient already had a quality colonoscopy (indicated by a withdrawal time of at least 6–9 minutes); therefore, it is reasonable to assume that no large lesion would have been missed. We suggest that we manage such cases conservatively with hydration, pain management, and close observation for at least the first 24 hours with the use of CT to monitor for resolution. Should there be a failure to resolve with conservative management, then it may be prudent to follow-up with a colonoscopy because this has been shown to reduce some cases of intussusception.^13^ Although we do not have considerable evidence regarding the effects on intussusception, it may be ideal to use carbon dioxide instead of room air as much as possible during insufflation because carbon dioxide will dissipate across the colon, causing fewer distension issues. Resection of intussuscepted bowel in these cases should be contemplated if patients' condition remains unchanged after the aforementioned conservative measures. Considering that colonoscopy-induced intussusception can be self-limiting, there may be a gross under-reporting of such cases. There is a need to investigate this issue further to develop more appropriate guidelines for managing colonoscopy-induced intussusception.

## DISCLOSURES

Author contributions: A. Ahmed wrote the manuscript. J. Zhang and K. Anas edited the manuscript. K. Anas is the article guarantor.

Financial disclosure: None to report.

Informed consent was obtained for this case report.
